# Psychological disorders and their influence on the development of periodontal disease in adolescents. A systematic review

**DOI:** 10.4317/medoral.27999

**Published:** 2026-01-24

**Authors:** Blanca Alagarda-Lauwers, Lucía Peñarrubia-Martínez, Marina García-Selva, Eva González-Angulo, Ana Cases-Sánchez, Carla Fons-Badal

**Affiliations:** 1 Dentist, private practice, Spain; 2 Department of Oral Medicine, Faculty of Medicine and Dentistry, University of Valencia, Spain

## Abstract

**Background:**

Periodontal disease is among the most prevalent oral conditions worldwide. Psychological disorders, typically diagnosed during the first two decades of life, have gained greater importance in recent years. Although the relationship between both conditions has been briefly studied, findings remain inconsistent. The objective of this systematic review is to analyze the involvement of psychological disorders on the development and progression of gingivitis and periodontitis.

**Material and Methods:**

This systematic review followed PRISMA guidelines and was registered in the PROSPERO (CRD420250651846). Searches were performed in PubMed, Scopus, Embase, and Cochrane Library using the equation: "(periodontal disease) AND ((adolescents) OR (teenager)) AND (psychological disorder)". Studies conducted in animals, adults, or with unrelated disorders were excluded

**Results:**

A total of 17 articles met the inclusion criteria. Adolescents with psychological disorders consistently showed poorer levels of periodontal health compared to controls, sharing several common risk factors among the pathologies.

**Conclusions:**

The general trend reflects a greater predisposition and prevalence of periodontal pathology symptoms in subjects with psychological disorder. Despite the promising findings, further research using standardized methodologies is required.

## Introduction

Periodontal disease is one of the most frequent oral conditions in the general population, with prevalence estimates ranging from 20% and 50%; therefore, this pathology is recognized as a public health problem (1). The American Academy of Periodontology states that in children and adolescents, periodontal diseases can appear with a different range of involvement, from gingivitis to some subtypes of periodontitis (2). Epidemiological, clinical, and histological studies have demonstrated that the development of gingivitis is age-related, with a progressive increase in prevalence and a peak during puberty (3 , 4). This pattern is closely linked to hormonal changes characteristic of the prepubertal and pubertal stages, which induce variations in the composition of the subgingival microflora (5). The association with systemic pathologies and the availability of effective and relatively simple treatments increase the importance of early treatment (6).

Certain systemic pathologies have been shown to be associated with both the onset and the progression of periodontal disease. The most widely studied synergies included diabetes, cardiovascular disease, obesity, rheumatoid arthritis, and chronic obstructive pulmonary disease (7). Additionally, the risk factors accompanying certain psychological disorders may also have a detrimental effect on periodontal pathology (8 , 9). Therefore, it is plausible to consider a potential relationship between neurological and psychological disorders and oral health (10).

This systematic review aims to determine whether psychological disorders act as a risk factors for the development and/ or severity of periodontal disease in adolescents, compared to their controls.

## Material and Methods

Study Protocol and Registration

This systematic review was conducted in accordance with PRISMA 2020. The protocol was prospectively registered in PROSPERO (CRD420250651846) following the standard structure (Population, Exposure, Comparator, Outcomes; eligibility criteria; search strategy; data extraction; risk of bias and synthesis plan). The protocol is available at the registration link: https://www.crd.york.ac.uk/PROSPERO/view/CRD420250651846

Search Strategy (Revised PICO Included)

The research question was defined using the PICO/PECO framework: Population (P): Adolescents (&lt;18 years); Exposure (E): Psychological disorders (ADHD, ASD, bipolar disorder, anxiety, depression); Comparator (C): Healthy adolescents; Outcomes (O): Periodontal indicators (GI, PI, CAL, PD, PDI, CPI).

A comprehensive electronic search was performed in February 2025 across PubMed/MEDLINE, Scopus, Embase, and The Cochrane Library (Cochrane Database of Systematic Reviews and Cochrane Central Register of Controlled Trials - CENTRAL). Searches were repeated at different time points to verify reproducibility of and ensure reliability.

The primary search string used in PubMed was:

( ("periodontal disease"[MeSH Terms] OR "periodontal disease" OR periodontitis) AND ((adolescent) OR (teenager)) AND ("psychological disorder" OR "mental disorder" OR "anxiety" OR "depression" OR "ADHD" OR "autism spectrum disorder" OR "bipolar disorder") ). A full search strategy for all databases were uploaded to PROSPERO. The PDF may be accessed through this link https://www.crd.york.ac.uk/PROSPEROFILES/67ea9c6999a4cf082eb6ed2171d6438e.pdf. No restrictions were applied during regarding publication year, study design, language, or publication status.

Selection and eligibility criteria

Only primary observational studies were included in the final synthesis. Due to the scarcity of evidence in adolescents, secondary studies (systematic reviews and meta-analyses) were consulted solely for contextual purposes but were not included as evidence, evaluated for risk of bias, or used in the final synthesis.

A detailed list of excluded full-text articles, including reasons for exclusion, will be provided in Supplementary Material (http://www.medicina.oral.com/carpeta/suppl1_27999).

Eligible studies included humans participants under 18 years of age (adolescent population) diagnosed with at least one of the following psychological disorders: Attention Deficit Hyperactivity Disorder (ADHD), Autism Spectrum Disorder (ASD), Bipolar Disorder, Anxiety, or Depression. Excluded studies involved animals, adults, or disorders outside the defined scope.

Included studies were required to assess at least one periodontal health parameter, such as Clinical Attachment Loss (CAL), Probing Depth (PD), Gingival Index (GI), Periodontal Disease Index (PDI), or Community Periodontal Index (CPI).

Data collection and extraction

**Figure 1:**
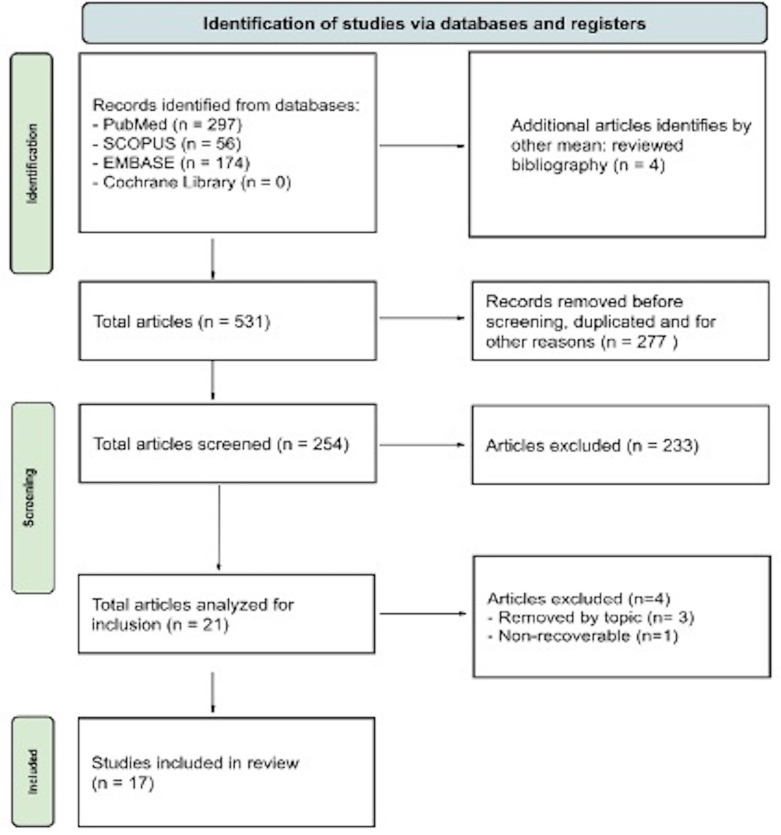
PRISMA flow diagram.

Two blinded reviewers independently conducted the selection process. After removing duplicates with EndNote software, titles and abstracts were screened according to the eligibility criteria. Full texts were then retrieved for final evaluation (Figure 1).

1

Data extraction from the included studies was performed independently by two reviewers using a standardized, pre-piloted form developed in Microsoft Excel. Extracted data included: Study characteristics (authors, year, design, sample size, country); population characteristics (age, gender, diagnosed psychological disorder); outcome measures (periodontal indices used) and key findings on the association (Effect measures, p-values, 95% CIs). Sources of heterogeneity were examined to the fullest extent permitted by the available data by ensuring comparable age ranges and by analyzing each disorder separately.

Risk of bias assessment &amp; publication bias

Publication bias could not be formally assessed via funnel plots due to heterogeneity and absence of meta-analysis; however, qualitative evaluation suggested that the observed pattern of results is unlikely to be attributable to selective publication.

Two reviewers independently assessed methodological quality. Cohort and case-control studies were evaluated using the Newcastle-Ottawa Scale (NOS), while cross-sectional studies were assessed with the JBI Critical Appraisal Checklist or the adapted NOS. Results of the quality assessment (risk of bias) are reported in detail, including a table summarizing the score for each domain for every included study. Only studies with low or moderate risk of bias were included in the final synthesis, typically scoring between 7 and 9 out of 9 on the NOS. This corresponds to a moderate-to-high certainty of evidence based on GRADE equivalence principles. Furthermore, the heterogeneity of the results, both positive and inconclusive, among the included studies does not indicate the presence of publication bias. Data synthesis and analysis Given the expected variability across study designs and periodontal indices, the primary synthesis was qualitative/narrative. A meta-analysis was planned only if sufficient homogeneity emerged. Depending on the study design, effect measures included Odds Ratios (OR), Relative Risks (RR), or mean differences.

## Results

A total of 17 studies met the eligibility criteria. These were grouped into 4 tables, each corresponding to a specific psychological disorder (Tables 1-4).

Table 1 Table 2 Table 3 Table 4

Results of the quality assessment (risk of bias) are reported in detail, including a table summarizing the score for each domain for every included study (Tables 5-7).

Table 5 Table 6 Table 7

A formal meta-analysis was not conducted due to the substantial heterogeneity across studies, including differences in periodontal diagnostic criteria, measurement indices, and follow-up periods. Pooling these data would have compromised statistical validity, resulting in an analysis lacking both consistency and statistical power; therefore, a qualitative descriptive synthesis was performed.

Attention Deficit Hyperactivity Disorder (ADHD)

Studies consistently reported higher levels of gingival bleeding among adolescents with ADHD compared with controls (11 - 13). A cohort study observed a significantly increased risk of periodontitis in the ADHD group (15.0 vs. 7.9 per 10,000 person-years), with earlier onset as well (1). However, a meta-analysis found no significant differences in the overall periodontal disease prevalence or plaque index scores (8).

Autism Spectrum Disorder (ASD)

Primary studies consistently showed poorer gingival health in adolescents with ASD. Case-control data reported significantly higher levels of gingival inflammation and greater periodontal treatment needs in the ASD group compared with controls (15). Yakubova et al. found markedly elevated PMA scores and a higher likelihood of affected periodontium in adolescents with ASD (16). These findings were supported by El Khatib et al., who observed significantly higher GI and PI values in ASD participants (19). Additionally, a descriptive study reported a high prevalence of gingivitis across dentitions in this population (17). Overall, evidence indicates increased gingival inflammation and periodontal susceptibility in adolescents with ASD (15 - 17 , 19).

Depression and anxiety

The results in this area were heterogeneous. One study identified no statistically significant association between gingivitis and psychological factors, although a positive trend was observed (20). Conversely, another study reported a significant association between moderate/severe gingivitis and depression (p=0.04), where adolescents with mild depression had twice the risk (OR=2.05) of developing moderate/severe gingivitis compared with healthy controls (10).

Bipolar disorder

Only one large cohort study (21,255 participants) was selected. Adolescents with bipolar disorder showed a significantly higher incidence of periodontitis than the control group (38.3% vs 14.7%), with an OR of 2.13. Long-term use of mood stabilizers further increased the risk of periodontitis (21).

## Discussion

The primary goal of this systematic review was to clarify the complex association between periodontal pathology and a range of psychological disorders in the adolescent population. Overall, the evidence indicated a consistent trend: Adolescents affected by psychological disorders exhibit a higher prevalence and greater susceptibility to gingivitis and periodontitis. This result requires a critical synthesis of the evidence, especially given the heterogeneity of the findings.

The methodological heterogeneity across included studies, particularly differences in periodontal diagnostic criteria, psychological disorder definitions, and measurement indices, limits comparability. This heterogeneity prevented meta-analysis and required narrative synthesis, consistent with PRISMA.

Secondary studies were reviewed only for contextual background, while primary evidence exclusively informed the final synthesis.

In the case of Attention-Deficit/Hyperactivity Disorder (ADHD), most studies reflected an increase in markers of periodontal pathology (1). Regarding the Bleeding Index, most investigations agree that the prevalence is higher in the affected group compared to controls (3 , 4 , 8 , 11 - 13). This elevated risk has been largely attributed to behavioral characteristics of ADHD-particularly hyperactivity, impulsivity, and reduced attention-which impair the maintenance of effective oral hygiene routines (12). Nevertheless, other studies did not report significant differences in plaque accumulation between ADHD and control groups (13 , 22), highlighting the variability in findings.

A critical exogenous factor influencing oral health across multiple psychological disorders is the impact of pharmacological treatments. Medications frequently prescribed for these conditions can produce side effects such as gingival enlargement, bruxism, and xerostomia. Xerostomia in particular is a major contributor to increased susceptibility to caries and periodontal disease (11). In individuals with Autism Spectrum Disorder (ASD), these effects may be compounded by sensory hypersensitivities, aversion to hygiene routines, behavioral challenges, and resistance to both professional and at-home dental care (23 , 24 , 25). Additional medication used by patients with ASD often exacerbates dry mouth and further compromises oral health (26 - 28).

For more severe mood disorders-particularly bipolar disorder-the mechanisms associated with periodontal vulnerability appear to extend beyond behavioral and pharmacological influences. Studies suggest the involvement of systemic alterations, including dysregulation of the hypothalamic-pituitary-adrenal axis that affects the immune response and the oral microbiome (29 - 31). These systemic factors may be further aggravated during depressive episodes by reduced motivation for self-care and oral hygiene.

The evidence regarding depression and anxiety remains inconsistent. While some studies found no statistically significant association with periodontal inflammation (10 , 32), others reported increased periodontal risk in individuals with symptoms of depression or dental anxiety (20 , 33 , 34). Many of these studies, however, involve adult populations, making it essential to interpret these findings with caution when applying them to adolescents. This variability highlights the necessity for careful comparison with prior literature and emphasizes the importance of establishing stronger, age-specific evidence.

A major strength of the present review is its focus on adolescents, a developmental period for which research is notably limited despite the increasing prevalence of psychological disorders and their potential impact on oral health. By integrating findings across a range of conditions, the review provides a comprehensive overview of the current evidence landscape.

The most notable limitation is the considerable methodological heterogeneity across the included studies. Differences in periodontal diagnostic criteria, measurement indices, study designs, and sample characteristics restricted the ability to perform a meta-analysis. Additionally, recruiting pediatric or adolescent populations with these disorders who do not present other comorbidities presents inherent challenges, often resulting in smaller samples and reduced generalizability.

Given the increased susceptibility to periodontal disease, dental professionals should adopt proactive, interdisciplinary screening for adolescents diagnosed with psychological disorders. Preventive strategies should specifically address medication-induced xerostomia through enhanced oral hygiene instruction, saliva substitutes, and regular follow-up.

To strengthen future evidence, more longitudinal, prospective studies are needed, employing standardized periodontal measures and explicitly accounting for confounding variables such as medication use and comorbidity profiles. Such research is crucial to establishing clearer causal pathways between psychological disorders and periodontal outcomes in adolescents.

## Conclusions

Based on the synthesis of this systematic review, several conclusions can be drawn regarding the influence of psychological disorders on the development of periodontal disease in the adolescent population:

1. A consistent general trend indicates that adolescents with psychological disorders demonstrate a higher prevalence and predisposition to periodontal pathology (gingivitis and periodontitis) compared to their healthy counterparts.

2. The most robust associations were found in Attention-Deficit/Hyperactivity Disorder (ADHD), reflected primarily in elevated Bleeding Index values and a significantly increased risk associated with Bipolar Disorder.

3. The primary contributing factors identified are deficient self-care and the systemic impact of pharmacological treatments. Medication-induced xerostomia and gingival enlargement appear to play a central role in accelerating periodontal disease progression.

4. Dental health professionals must implement proactive, interdisciplinary screening protocols and tailored preventive strategies for this patient cohort, with particular focus on mitigating medication-related oral side effects.

5. Future investigations require a shift towards longitudinal, prospective study designs using standardized, objective periodontal indices to overcome the current methodological heterogeneity and establish clearer causal relationships in adolescents.

## Figures and Tables

**Table 1:** Table Data synthesis from selected studies on ADHD.

Author and year	Type of study	Periodontal disease	Measurement	Results
Drumond et al., 2022	Systematic review and meta-analysis	Gingivitis and/or periodontitis	GBI, PI, Gingivitis	Greater bleeding in the ADHD group (MD=11.25; CI=0.08-22.41; I 2=73%).
Hsu et al., 2024	Cohort study(2001-2011)	Periodontitis	Taiwanese treatment codes	Significantly higher risk of periodontitis in the ADHD group (15.0) comparedto the control group (7.9) p<.001.Earlier onset (16.39+/-2.39) vs.(21.50+/-2.86 years, p<.001.
FernÃ¡ndez-Arce et al., 2024	Cross-sectional case-control study	Gingivitis	GBI (ESN-E 2017 data)	Gingival bleeding (5.1% vs. 8.2%) in children without/with ADHD.Adjusted OR: 1.64; 95% CI: 1.11-2.41.
Chau YC et al., 2017	Cross-sectional case-control study	Gingivitis	GBI, CPI, salivary samples and questionnaires	Mean percentage of gingivalbleeding significantly higher inchildren with ADHD.
Blomqvist M et al., 2011	Cross-sectional case-control study	Gingivitis	GBI, oral health habits questionnaire	Bleeding on probing: 35±39% (ADHD) vs 16±24% (controls).

1

**Table 2:** Table Data synthesis from selected studies on ASD.

Author and year	Type of study	Periodontal disease	Measurement	Results
Fakroon et al., 2014	Case-control study	Gingivitis and/or periodontitis	CPITN and OMS 1997 criteria (index teeth)	Greater treatment needs in ASD group, signs of gingival inflammation were present in almost twice as many participants as in the control group.
Yakubova et al., 2023	Cross-sectional case-control study	Gingivitis	PMA index (Papillary, Marginal and Attached gingiva).	OR: 3,7 affected periodontium in ASD vs. control group.Light gingivitis: 11,18±0,69 in ASD vs. 7,38±0,19 in the control group.Moderate gingivitis: 30,76±0,57 in ASD.
Vishnu Reckha et al., 2012	Cross-sectional study (without control group)	Gingivitis	Gingivitis	High rate of gingivitis in mixed and permanent dentition.
El Khatib et al., 2013	Cross-sectional case-control study	Gingivitis	Silness and Loe PI, GI	Statistically significant differences in GI between ASD and healthy children.

2

**Table 3:** Table Data synthesis from selected studies on anxiety and depression.

Author and year	Type of study	Periodontal disease	Measurement	Results
da Silva et al., 2014	Cross-sectional case-control study	Gingivitis	CPI, bleeding interview	Positive correlation between scores on the Revised Children's Manifest Anxiety Scale (RCMAS), the Children's Depression Inventory (CDI) and the presence of gingivitis.
Folayan et al., 2021	Cross-sectional case-control study	Gingivitis	Loe and Silness GI, PI in index teeth	Significant association between moderate or severe gingivitis, plaque index (P<0.0001) and depression (P=0.04).Patients with mild depression had an OR of 2.05, 95% CI of 1.12, 3.73, and a higher risk of having moderate/severe gingivitis compared to healthy adolescents.

3

**Table 4:** Table Data synthesis from the analyzed study on bipolar disorder.

Author and year	Type of study	Periodontal disease	Measurement	Results
Wu et al., 2023	Cohort Study	Periodontitis and gingivitis	ICD-9-CM codes, Taiwanese periodontitis treatment codes.	Periodontitis: 38.3% in bipolar group vs. 14.7% controls (OR=2.13),bipolar disorder was associated with a higher prevalence of gingivitis.Long-term mood stabilizers increased periodontitis risk (HR: 1.19; 95% CI: 1.06-1.35).

4

**Table 5:** Table Quality of studies on the Newcastle-Ottawa scale for case-control studies.

	Selection (xxxx)				Comparability (xx)	Exposure (xxx)			
Author-Year	Case definition adequate	Representativeness of cases	Selection of controls	Definition of controls	Comparability of cases & controls	Ascertainment of exposure	Same method of ascertainment for cases & controls	Non-response rate	Final score
Chau et al., 2017	x	x	x	x	xx	x	x	x	9 x
Blomqvist et al., 2011		x	x	x	xx	x	x		7 x
Fakroon et al., 2014	x		x	x	xx	x	x	x	8 x
Yakubova et al., 2023	x		x	x	xx	x	x		7 x
El Khatib et al., 2013	x	x	x	x	xx	x	x	x	9 x
da Silva et al., 2014	x	x	x	x	xx	x	x	x	9 x
FernÃ¡ndez-Arce et al., 2024	x	x	x	x	xx		x		7 x

5

**Table 6:** Table 6

	Selection (xxxx)				Comparability(xx)	Outcome (xxx)			
Author - Year	Representativeness of the exposed cohort	Selection of the non exposed cohort	Ascertainment of exposure	Demonstration that the outcome of interest was not present at start of study	Comparability of cohorts on the basis of design or analysis	Assessment of outcome	Duration of follow-up	Adequacy of cohort follow-up for cohorts	Final Score
Hsu et al., 2024	x	x	x	x	xx	x	x	x	9 x
Wu et al., 2022	x	x	x	x	xx	x	x	x	9 x

6

**Table 7:** Table 7

	Selection(xxxxx)				Comparability(x)	Outcome (xxx)		
Author - year	Sample representativeness	Sample size	Non-response rate	Exposure determination		Outcome evaluation	Statistical test	Final score
Vishnu- Reckha et al., 2012	x	x	x	x		xx	x	8 x
Folayan et al., 2021	x	x	x	x	x	xx	x	9 x

7

## Data Availability

Declared none.
